# Evaluation of the Correlation between Gut Microbiota and Renal Function in Chronic Kidney Disease Patients

**DOI:** 10.4014/jmb.2502.02039

**Published:** 2025-06-17

**Authors:** Jun Xi, Jiru Xu, Jujun Sun, Huan Li, Siruo Zhang, He Xie, Haining Wang, Rui Zhang, Meng Zhao, Mi Wang, Tian Zheng

**Affiliations:** 1 The XD Group Hospital, Xi’an 710077, Shaanxi Province, P.R. China; 2 Department of Microbiology and Immunology, Key Laboratory of Environment and Genes Related to Diseases of Chinese Ministry of Education, School of Medicine, Xi’an Jiaotong University, Xi’an 710061, P.R. China; 3 Xi’an Mental Health Center, Shaanxi Provincial Mental Health Center Laboratory, Xi’an 710061, P.R. China; 4 Shaanxi Provincial People’s Hospital, Xi’an 710068, P.R. China; 5 Department of Blood Transfusion, Xi’an No.3 Hospital, The Affiliated Hospital of Northwest University, Xi’an 710021, Shaanxi, P.R. China; 6 Department of Clinical Laboratory, The First Affiliated Hospital, Air Force Medical University, Xi’an 710032, P.R. China

**Keywords:** Chronic kidney disease, gut microbiota, 16S rRNA gene sequencing

## Abstract

Chronic kidney disease (CKD) has recently emerged as a significant, global public health threat, and the gut microbiota are now recognized as playing a crucial role in the pathogenesis and progression of CKD. In this study, we investigated the correlation between differential gut microbiota and renal function impairment by analyzing the structure, diversity, and other characteristics of the gut microbiota in patients with CKD. Our findings indicated that CKD patients exhibit decreased species diversity and evenness in their gut microbiota compared to healthy individuals, with notable differences in community composition between the groups. Among them, p_*Actinobacteriota*, p_Cyanobacteria, g_*Faecalibacterium*, g_*Agathobacter*, g_*Roseburia*, and g_*Actinomyces* were significantly decreased (*p* < 0.05), while p_Acidobacteriota, g_*Blautia*, and g_*Candidatus-Solibacter* were significantly increased in CKD (*p* < 0.05). Furthermore, functional prediction results suggested that the differential pathways primarily involved metabolic pathways, including Carbohydrate Metabolism, Glycan Biosynthesis and Metabolism, Biosynthesis of Other Secondary Metabolites, Metabolism of Other Amino Acids, and pathways related to Endocrine and Metabolic Diseases. Meanwhile, correlation studies revealed a significant negative correlation between g_*Actinomyces* and serum uric acid levels (*r* = -0.426, *p* = 0.038), and a significant positive correlation between g_*C. Solibacter* and serum uric acid levels (*r* = 0.461, *p* = 0.023). This study highlights the significant differences in the composition and species abundance of gut microbiota between CKD patients and healthy individuals, while also demonstrating that the abundances of g_*Actinomyces* and g_*C. Solibacter* are correlated with serum uric acid levels, an indicator of renal function impairment.

## Introduction

Chronic kidney disease (CKD) is a chronic illness that significantly impacts public health. With the intensification of global population aging and changing lifestyles, the prevalence of CKD has markedly increased worldwide [1]. Most patients do not exhibit obvious symptoms in the early stages of the disease, and are often diagnosed with abnormal kidney function during routine examinations. When kidney damage persists for more than 3 months, patients may experience a decline in glomerular filtration rate (GFR) and elevations in renal damage markers, such as serum creatinine (Scr), blood urea nitrogen (BUN), urine albumin-to-creatinine ratio (ACR), urine β_2_-microglobulin, and increased urine albumin-to-creatinine ratio [2]. As the disease progresses, patients may develop a range of complications including digestive system abnormalities, cardiovascular diseases, anemia, neurological symptoms, and even death [3]. However, there are many causes of CKD, including primary glomerulonephritis, diabetic nephropathy, and hypertensive nephropathy, among others, all posing challenges for the prevention and treatment of CKD [4, 5]. The high incidence and low awareness of CKD result in many patients missing the optimal timing for early detection and intervention, which are of utmost importance in delaying progression of this disease.

The Human Microbiome Project (HMP) has revealed that gut microbiota, which may outnumber human cells by a factor of 10, play a crucial role in human health and diseases including diabetes, obesity, rectal cancer, kidney disease, and more [6, 7]. In recent years, a growing number of studies have shown a close correlation between gut microbiota and CKD [8, 9]. Scientists using the 5/6 nephrectomy model in mice recently altered the gut microbiota and induced colonic inflammation, thereby changing gastrointestinal motility function and leading to constipation. This demonstrates a significant correlation between renal failure and gastrointestinal dysfunction caused by alterations in the gut microbiota [10]. Previous studies have shown that during the progression of CKD to end-stage renal disease (ESRD), protein-bound uremic toxins, such as indoxyl sulfate, p-cresol glucuronide, p-cresol sulfate, and indole-3-acetic acid, progressively accumulate. This accumulation is postulated to be associated with enhanced toxin production caused by gut microbiota dysbiosis and diminished toxin clearance due to kidney impairment. Furthermore, it may be accompanied by the onset of intestinal inflammation and epithelial barrier dysfunction, resulting in accelerated systemic translocation of bacterial-derived uremic toxins, ultimately causing oxidative stress damage not only to the kidneys, but to the cardiovascular and endocrine systems as well [11]. However, the mechanism by which gut microbiota contribute to the progression of CKD remains unclear. Therefore, we sought to analyze the correlation between changes in gut microbiota and renal function markers in patients with CKD, with the goal of providing basic data for exploring the pathogenesis of CKD from the perspective of gut microbiota.

## Materials and Methods

### Sample Collection

Forty-two patients with CKD were recruited as the experimental group, and 42 age- and gender-matched healthy volunteers were randomly selected as the control group (Table 1). The enrolled patients spanned CKD stages 1-5 (stages 1-4: 22; stage 5: 20, with no significant differences in stage distribution between subgroups (*p* > 0.05)). The diagnostic criteria for CKD adhered to the guidelines proposed by the Kidney Disease Outcomes Quality Initiative (KDOQI) of the National Kidney Foundation (NKF), the Kidney Disease Improving Global Outcomes (KDIGO), and the Chinese Society of Nephrology: kidney structure and function abnormalities persisting for more than 3 months, regardless of the cause. Specifically, this included pathological damage to the kidney, abnormalities in blood or urine composition, abnormalities in imaging examinations, or a glomerular filtration rate (GFR) less than 60 mL/min/1.73 m² for over 3 months [12]. All participants resided in the city or surrounding counties and shared similar dietary habits. None of the subjects had mental or gastrointestinal diseases, and they had not used antibiotics, probiotics, or other preparations that could affect gut microbiota within 1 month prior to sample collection. Additionally, the CKD group had not undergone dialysis treatment. All participants provided written informed consent, and the study was approved by the Ethics Committee of the XD Group Hospital (No. 20240910-01).

Participants were instructed to collect a morning fecal sample by utilizing a sterile swab to obtain 1 g of feces and deposit it into a capped, sterile container. Fasting whole blood was collected from seated participants in the morning using an EDTA blood collection tube. The blood samples were centrifuged at 3,000 rpm for 30 min to promptly separate the plasma, which was subsequently transferred to 1.5 ml centrifuge tubes. All samples were then transported at low temperatures using adequate dry ice and stored in a -80°C freezer.

### Serum Renal Function Testing

The renal function indicators of two groups of serum samples, including serum creatinine (Cr), blood urea nitrogen (BUN), uric acid (UA), cystatin C (Cys C), β2-microglobulin (β2-MG) and other serum nutritional indicators were tested using the fully automated Roche Cobas C501 Chemistry Analyzer (Roche Diagnostics GmbH, Switzerland) with corresponding reagents. The glomerular filtration rate (GFR) was also calculated.

$$\text{GFR}\left(\text{ml/min/1}{\text{.73m}}^{\text{2}}\right)\text{=186×}{\left(\frac{\text{Serum Creatinine }\left(\text{μmol/l}\right)}{\text{88.4}}\right)}^{\text{-1.154}}{\text{×Age}}^{\text{-0.203}}\text{×Coefficient}$$ (1)

### Extraction of Gut Microbiota

Bacterial genomic DNA was extracted from 200 mg of fecal samples using the TIANamp Stool DNA Kit DP328 (Tiangen Biotech, China) according to the manufacturer's instructions. The concentration of the extracted DNA was measured using a NanoDrop 2000 Spectrophotometer (Thermo Fisher Scientific, USA) to ensure it met the quality standards, and then stored in a -80°C freezer.

### 16S rRNA High-Throughput Sequencing

In this study, universal primers targeting the V3 region of the 16S rRNA gene were utilized for sequencing analysis and species identification within the hypervariable region. The primer sequences were V3-341F (5'-CCTACGGGAGGCAGCAG-3') and V3-534R (5'-ATTACCGCGGCTGCTGG-3'), synthesized by Sangon Biotech (China). Each PCR mixture comprised 15 μl of Phusion High-Fidelity PCR Master Mix, 0.2 μM of each primer, and 10 ng of genomic DNA template. PCR amplification was initiated with denaturation at 98°C for 1 min, followed by 30 cycles of 98°C for 10 s, 50°C for 30 sec, and 72°C for 30 sec, with a final extension at 72°C for 5 min. Magnetic bead purification was performed on the PCR products, and equal amounts of the products were mixed based on their concentrations. After thorough mixing, the qualified PCR products were sequenced on the NovaSeq 6000 (Illumina, USA). Following the assembly and filtering of reads, the effective data (clean data) obtained from operational taxonomic unit (OTU) clustering were annotated for species and analyzed for abundance. Then, through alpha diversity, beta diversity analysis, principal component analysis (PCA), and non-metric multi-dimensional scaling (NMDS), we obtained information on species and differences in community structure among samples. Finally, functional annotation information was predicted using the Tax4Fun function.

### Statistical Methods

Statistical analysis of the serum data was performed using SPSS 19.0 software. Chi-square tests were used for categorical data. For quantitative data, *t*-tests were applied to compare two independent samples that followed a normal distribution, while the Wilcoxon rank-sum test was used for those that did not. Spearman's rank correlation test was chosen for correlation analysis. A *p*-value of < 0.05 was considered statistically significant. QIIME software (Version 1.9.1) was used to calculate the Observed-OTUs, Chao1, Shannon, Simpson, ACE, and PD_whole_tree indices. R software (Version 2.15.3) was utilized for analyzing differences in alpha and beta diversity indices between groups, with *t*-tests and Wilcoxon tests applied to the two sample groups. PCA analysis was conducted using the ade4 and ggplot2 packages in R, while NMDS analysis was performed using the vegan package in R.

## Results

### Clinical Data and Serological Test Results

In this study, *t*-tests or Wilcoxon rank-sum tests were used to analyze clinical renal function indicators. The results showed significant differences in serum renal function indicators between the two groups. The GFR, BUN, CREA, UA, CyC, and serum β2-MG levels were significantly higher in the CKD group compared to the control group, indicating marked impairment of renal function in the CKD group (*p* <0.001) (Table 2). Additionally, serum levels of potassium (K), triglycerides (TG), glucose (GLU), and Apolipoprotein B (APOB) were significantly higher in the CKD group compared to the control group (*p* <0.05), while serum levels of calcium (Ca), total protein (TP), albumin (ALB), and whole blood hemoglobin (HB) were significantly lower (*p* <0.05) (Table 1).

### Sample Sequences and OTU Number Statistics

Based on preliminary experimental findings and resource optimization considerations, we employed computer-generated randomization to select 12 cases per group from the original 42-subject pool for 16S rRNA gene sequencing using the Illumina Nova platform. This approach ensured: (1) equal inclusion probability across all participants to maintain demographic/clinical heterogeneity inherent to the parent cohort; (2) rigorous minimization of selection bias through algorithm-driven impartiality; and (3) technical feasibility by balancing sequencing depth (tens of thousands of reads per sample) with cost-effectiveness, while preserving statistical power for population-level microbial signature inference. Following sequencing, a library was constructed for operational taxonomic unit (OTU) clustering, resulting in a total of 2,568,153 raw sequences. After chimera filtering, 1,581,490 valid sequences were retained, with a total of 657,128,708 bases. The average number of valid sequences per sample was 65,895 ± 3,079, and the average sequence length was 415 ± 3 bp (Table 3). A total of 1,496 OTUs were identified, of which 1,491 (99.67%) could be annotated to the database. Annotation rates were 94.65%at the phylum level, 94.12% at the class level, 90.11% at the order level, 79.88% at the family level, and 58.96% at the genus level.

### Analysis of Intestinal Flora Structure

**Fig. 1:**
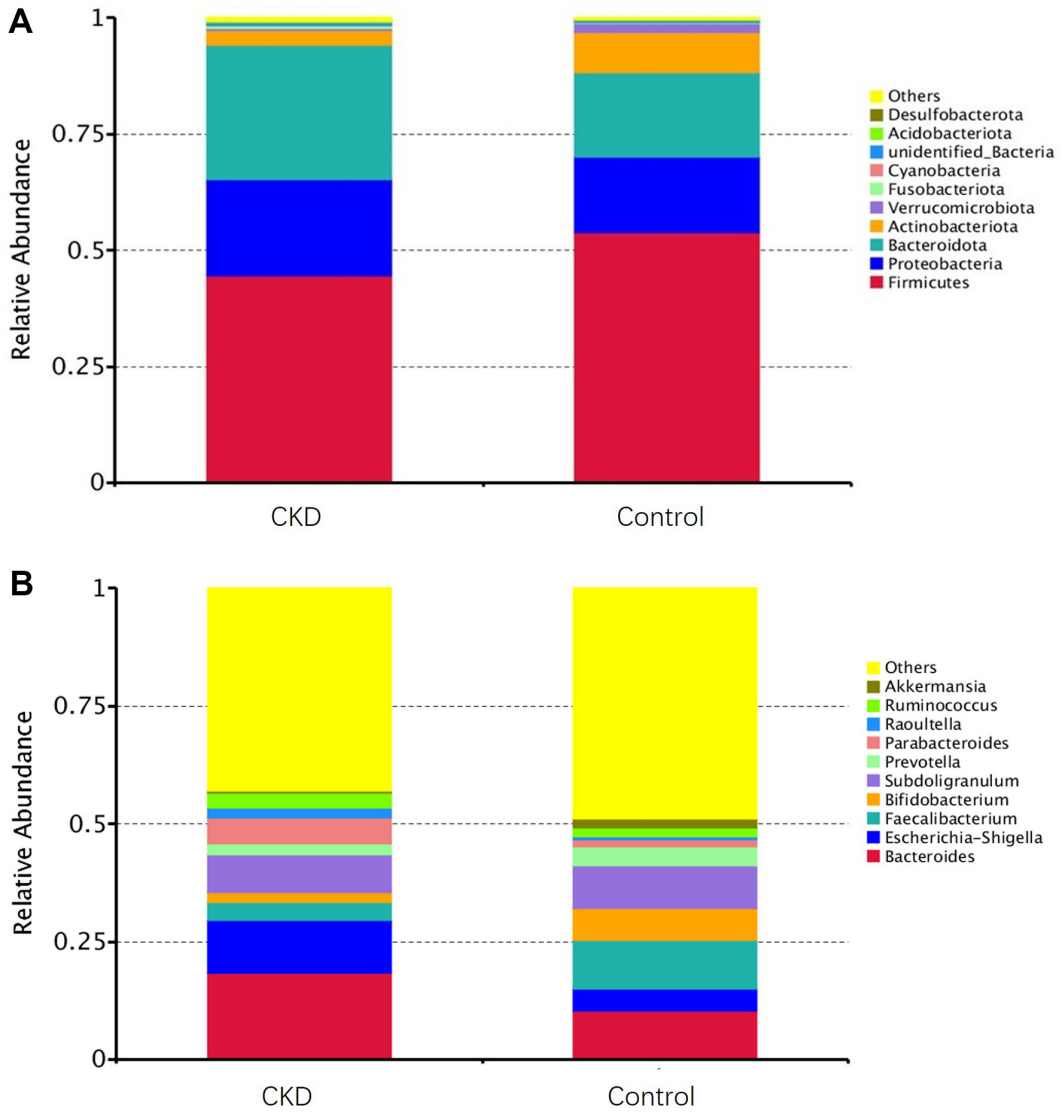
Bar graph of relative abundance of species in intestinal flora. (**A**) The top 10 bacterial phylum; (**B**) The top 10 bacterial genus. The abscissa is the group, the ordinate is the relative abundance, and different colors represent different bacterial groups.

Based on the microbiota annotation results, the species abundance of the intestinal flora in the two groups of samples was analyzed in terms of phylum and genus. The optimal species in the two groups of samples are Firmicutes and Bacteroidota, with higher abundance of Proteobacteria and Actinobacteria. The species diversity of the gut microbiota at the phylum level in patients with CKD shows no significant difference compared to that in healthy individuals, but the relative abundance has changed. Specifically, the abundance of Firmicutes and Actinobacteria is lower in CKD patients compared to healthy individuals, while the abundance of Bacteroidota and Proteobacteria has increased (Fig. 1A). The top 10 genera in the CKD group were *Bacteroides*, *Escherichia-Shigella*, *Subdoligranulum*, *Parabacteroides*, *Faecalibacterium*, *Blautia*, *Ruminococcus*, *Klebsiella*, *Prevotella*, and *Raoultella*, while the top 10 genera in the control group were *Bacteroides*, *Faecalibacterium*, *Subdoligranulum*, *Bifidobacterium*, *Klebsiella*, *Escherichia-Shigella*, *Prevotella*, *Streptococcus*, *Dialister*, and *Ruminococcus*. In addition, *Faecalibacterium* and *Blautia* increased in the CKD group, while abundances of *Agathobacter* and *Roseburia* were lower than those in the control group (Fig. 1B).

### Analysis of Microbial Diversity and Differential Species

**Fig. 2:**
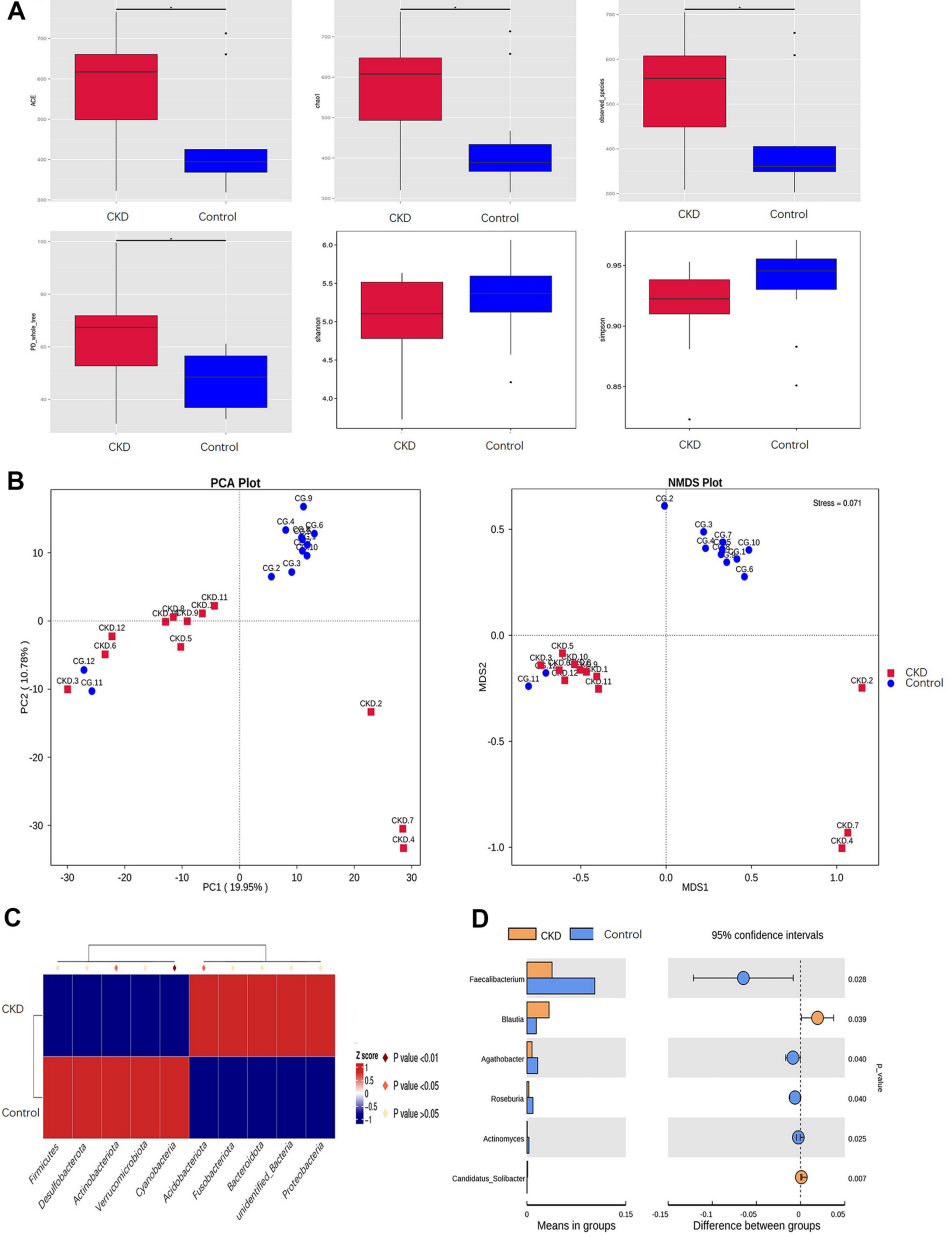
Species diversity analysis and differential species. (**A**) α diversity analysis index. Observed_species: The number of species that are visually observed, also known as the number of OTUs. Shannon: The total number of categories in the sample and their proportion. Simpson: It characterizes the diversity and evenness of species distribution within a community. Chao1: Estimate the total number of species contained in the community sample. ACE: estimate the number of OTU in the community. PD_whole_tree: The phylogenetic relationships among species within the community. (**B-D**) β diversity analysis. (**B**) Principal Component Analysis (PCA): The x-axis represents the first principal component, with the percentage indicating its contribution to the sample variance; the y-axis represents the second principal component, with the percentage indicating its contribution to the sample variance. Each point on the plot represents a sample, and samples from the same group are represented by the same color. Closer distances between samples on the PCA plot indicate more similar community compositions. Non- Metric Multi-Dimensional Scaling (NMDS): Each point represents a sample, and the distance between points indicates the degree of difference. Samples from the same group are represented by the same color. A Stress value < 0.2 indicates that the NMDS accurately reflects the degree of difference between samples. (**C**) MetaStat heatmap for significant differences in species at the phylum level. MetaStat was used to perform between-group differential analysis of gut microbiota at the phylum level, with statistical significance defined as *P* < 0.05. (**D**) T-test plot for significant differences in species at the genus level. For genuslevel analysis, *t*-tests were applied to identify differences between groups. The left bars represent the abundance of differential genera across groups, while the right bars indicate the significance levels. The *p*-value represents the probability value, with *P* < 0.05 considered statistically significant. *P* < 0.05 is marked with *; *P* < 0.01 is marked with **; *P* < 0.001 is marked with ***.

As shown in Figs. A1-A3 in the appendix, this study has sufficient sampling and reasonable sequencing data. OTUs and α-diversity indices ACE, Chao1, PD_whole_tree, Shannon and Simpson were used to evaluate species richness, evenness, and diversity. The Shannon index in the CKD group was lower than that in the control group and the difference was not statistically significant (CKD 5.028 vs. control 5.315, *p* = 0.22), and the same followed for the Simpson index (CKD 0.916 vs. control 0.935, *p* = 0.20), suggesting that the species diversity and evenness of the CKD group were reduced compared to the control group, but the difference was not significant. Meanwhile, the Chaol, ACE, and PD_whole_tree indices showed a significant increase (*p* = 0.03, *p* = 0.02, *p* = 0.02), indicating that the number of species in the CKD group was significantly higher than that in the control group, and the relationship was closer (Fig. 2A). Further PCoA analysis based on Unifrac distance and NMDS analysis based on Bray-Curtis distance showed that the distance between samples within the group was close, while the distance between groups was far, indicating that the community composition within the group was similar, while the community composition between groups was different (Fig. 2B). The Anosim and Adonis analysis results of the significant difference test between groups showed that the R-value was between -1 and 1 and greater than 0, with *p* < 0.05, indicating that there was a significant difference between the CKD group and the control group (Table 4).

Further analysis using MetaStat was conducted to identify species that differed between the two groups at the phylum level. The results showed that Actinobacteriota, Cyanobacteria, and Acidobacteriota were significantly different between the two groups, with Actinobacteriota and Cyanobacteria significantly decreased in CKD (*p* < 0.05), while Acidobacteriota significantly increased in CKD (*p* < 0.05) (Fig. 2C). Using *t*-test analysis to identify species that differed at the genus level, six bacterial groups were found to be significantly different, including *Faecalibacterium* (*p* = 0.028), *Blautia* (*p* = 0.039), *Agathobacter* (*p* = 0.040), *Roseburia* (*p* = 0.040), *Actinomyces* (*p* = 0.025), and *Candidatus Solibacter* (*p* = 0.007). Among them, *Blautia* and *C. Solibacter* had varying degrees of increase in abundance in the CKD group (Fig. 2D).

### Network and Function Analysis

**Fig. 3:**
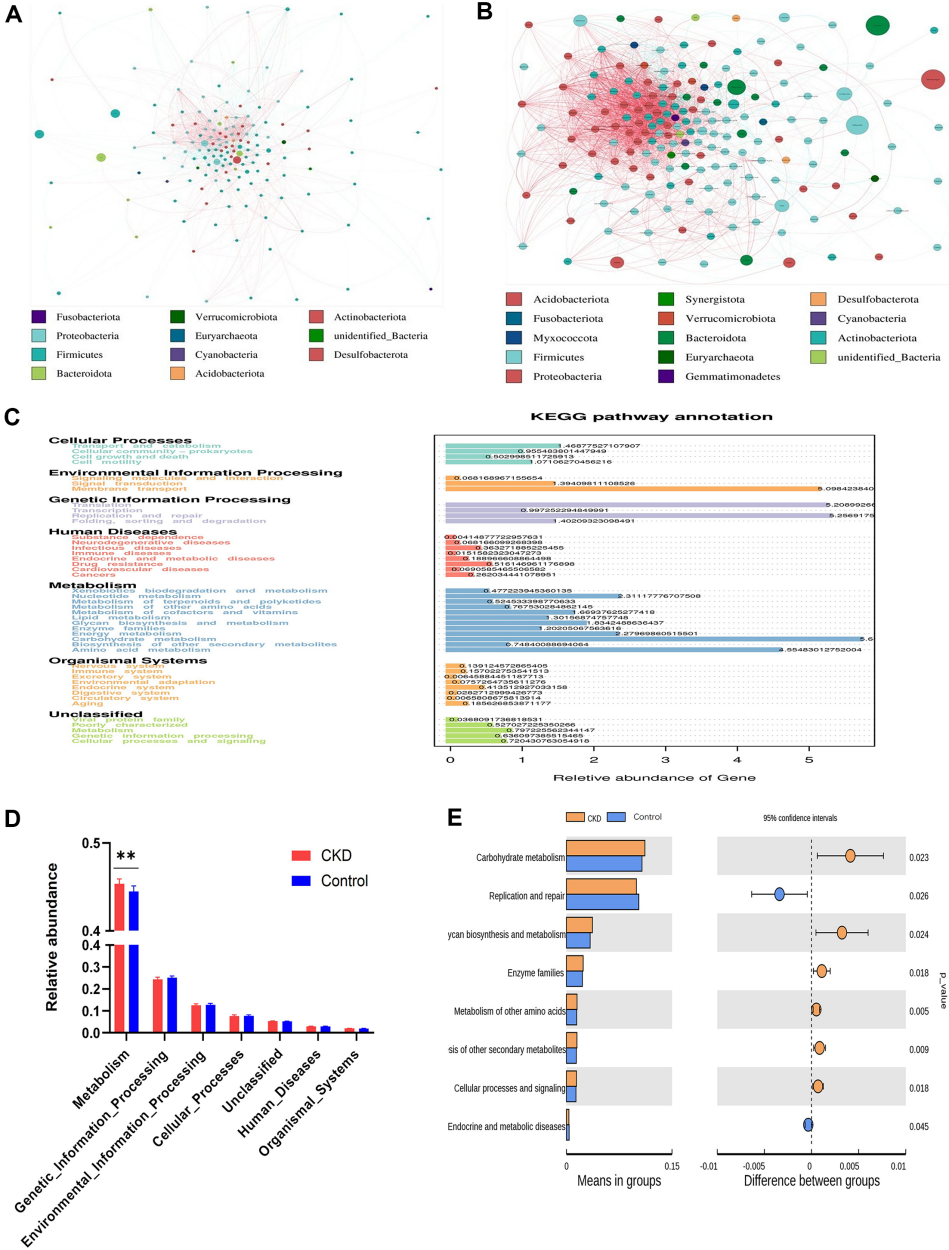
Network and function annotation. (**A**) CG group Network. (**B**) CKD group Network. Different nodes represent different genera, and the size of the node represents the average relative abundance of the genus. The nodes of the same phylum have the same color, and the thickness of the lines between nodes is positively correlated with the absolute value of the correlation coefficient between species interactions, with red indicating positive correlation and blue indicating negative correlation. (**C**) Statistical chart of KEGG database prediction results. The numbers on the bar chart represent the number of genes predicted by the Tax4Fun function. The horizontal axis represents the number of predicted genes, and the vertical axis represents the description of gene functions at the first and second levels. (**D**) T_test analysis diagram of functional gene annotation at L2 level. The left figure shows the average relative abundance of gene function annotation, and the right figure shows the confidence level of inter-group differences. The leftmost point of each circle in the figure represents the lower limit of the 95% confidence interval for the mean difference, and the rightmost point of the circle represents the upper limit of the 95% confidence interval for the mean difference. The center of the circle represents the difference in the mean values. The group represented by the color of the circle is the group with a high mean value. The rightmost part of the display results is the *p*-value of the inter-group significance test for the corresponding function, *P* < 0.05 was considered statistically significant.

A co-occurrence network diagram was constructed based on the Spearman correlation index *r* of the two groups of samples. The results showed that the vast majority of bacterial genera in both groups were from the Firmicutes phylum. Compared with the control group, the genera with higher relative abundance in the CKD group were *Bacteroides*, *Escherichia-Shigella*, and *Subdoligranulum*. Among them, *Escherichia-Shigella* was positively correlated with *Eubacterium_ventriosum*_group and *Anaerostipes*, while *Bacteroides* was positively correlated with *Faecalibacterium*, but negatively correlated with *Weissella*. In addition, The CKD group samples had significantly higher network complexity (Fig. 3A and 3B).

To investigate the relationship between gut microbiota and host metabolic changes, we conducted a Tax4Fun functional analysis. We obtained 5,878 KEGG pathways from 16,329 KEGG gene sequences, with the vast majority of genes corresponding to the metabolic pathways of metabolism. The top five metabolic pathways with the greatest differences were carbohydrate metabolism, glycan biosynthesis and metabolism, nucleotide metabolism, lipid metabolism, and amino acid metabolism (Fig. 3C). Level 1 analysis revealed significantly elevated gene expression in metabolic pathways in the CKD group (*p* = 0.002) (Fig. 3D). Level 2 identified six gene categories that were significantly more abundant in the CKD group compared to controls, including carbohydrate metabolism (*p* = 0.023), glycan biosynthesis and metabolism (*p* = 0.024), enzyme families (*p* = 0.018), biosynthesis of other secondary metabolites (*p* = 0.009), metabolism of other amino acids (*p* = 0.005), and cellular processes and signaling (*p* = 0.018). Conversely, replication and repair (*p* = 0.026) and endocrine and metabolic diseases (*p* = 0.045) were significantly less represented in the CKD group than in controls (Fig. 3E). Specifically, Tax4Fun analysis suggested differential metabolic pathway profiles between CKD patients and controls, yet the accuracy of these predictions requires verification through metagenomic sequencing and metabolomics studies.

### Correlation between Serum Renal Function and Gut Microbiota Species Differences

Using SPSS, we conducted a Pearson correlation test between bacterial genera with significant differences and serological markers of renal function. The results indicated that Actinomyces was significantly negatively correlated with serum uric acid levels (*r* = -0.426, *p* = 0.038), whereas *C._Solibacter* was significantly positively correlated with serum uric acid levels (*r* = 0.461, *p* = 0.023) (Table 5). Compared to healthy individuals, patients with CKD exhibited increased levels of blood uric acid, decreased abundance of *Actinomyces*, and increased abundance of *C. Solibacter*.

## Discussion

The results of this study suggest that there may be an interaction between serum renal function injury and gut microbiota in patients with CKD. In recent years, scientists have collated a total of 980 samples from six references in the PubMed, Web of Science, and GMrepo databases from three countries, and the 16S rRNA microbiome data were processed by DADA2 QIIME2 and PICRUSt2 analysis. The results revealed that the intestinal flora of CKD patients was significantly different from that of the healthy control (HC), and the microbial diversity in the CKD group was noticeably reduced. Furthermore, the abundance of *Faecalibacterium prausnitzii* in the CKD group was significantly reduced as detected by the linear discriminant analysis effect size LEfSe analysis [13]. Previous study has shown that the abundance of [*Ruminococcus*] *gnavus* group is significantly increased in patients with early-stage CKD combined with hyperuricemia [14]. Emerging evidence proves that intestinal flora plays an important role in CKD. The subtle relationship between the gut-kidney axis may not only be dominated by bacterial effects, but may also involve parasites. A recent Thai study suggested that infection with *Strongyloides stercoralis* can induce intestinal dysbiosis in patients with CKD. SCFA-producing bacteria, such as anaerobes and Coprococcus_1, were significantly reduced in the CKD group infected with *S. stercoralis*, while the genera *Escherichia-Shigella* and *Anaerostipes* showed an opposite trend [15]. These results suggest that there may be a correlation between gut microbiota and CKD.

We studied the changes in the structure of the gut microbiota in patients with CKD, and the results showed that the species diversity and evenness of the gut microbiota in the CKD group decreased. The top three species of gut microbiota at the phylum level that dominate the intestinal tract of patients with CKD include Firmicutes, Bacteroidota, and Proteobacteria, while Actinobacteriota and Cyanobacteria are significantly reduced in the CKD group, whereas Acidobacteriota is significantly increased. Abundances of the genera *Faecalibacterium*, *Agathobacter*, *Roseburia*, and *Actinomyces* decreased, while *Blautia* and *C. Solibacter* increased to varying degrees. Previous studies have shown that there are many factors that can affect the changes in intestinal flora. The gut microbiota undergoes extensive changes throughout life, and age-related processes may affect the gut microbiota and its associated metabolic changes. Research indicates that compared to the young, the α-diversity of microbial taxa, functional pathways, and metabolites in the elderly is higher, particularly in the oldest individuals. The β-diversity distance varies significantly at different developmental stages, even between the most elderly and younger adults. With aging, *Akkermansia* is relatively abundant, while the relative abundance of Firmicutes, Bacteroidetes, and *Lactobacillaceae* decreases. At the same time, pathways related to carbohydrate metabolism and amino acid synthesis decrease in elderly people [16]. The use of antibiotics can have various negative effects on the gut microbiota, including reduced species diversity, altered metabolic activity, and selection of antibiotic-resistant microorganisms, which in turn can lead to antibiotic-associated diarrhea and recurrent *Clostridium difficile* infection [17]. Research has shown that treatment with the anti-metabolism drug 5-Fluorouracil can also markedly reduce the number of Actinobacteria in the mouse gut and change the abundance of *Enterobacteriaceae*, *Lactobacillaceae* NK4A136 group, *Escherichia-Shigella*, *Bacteroides*, *Lactobacillus*, *Alphaproteobacteria*, *Rikenellaceae*, and *Bordetella* [18]. Research has also demonstrated that the gut microbiota of mice exposed to smoke is dysregulated, with significant differences in the abundance of bacterial species, including enrichment of *Eggerthella lenta* and depletion of *Parabacteroides distasonis* and *Lactobacillus* spp. [19]. In this study, we found that a long-term, low-protein diet in patients with CKD may change the level of uremic toxins in the digestive environment, which may affect the composition of the gut microbiota [20].

The functional prediction results showed that the differential pathways were mainly concentrated in metabolic pathways. Previous studies have shown that the top five metabolic pathways with the greatest differences are carbohydrate metabolism, glycan biosynthesis and metabolism, nucleotide metabolism, lipid metabolism, and amino acid metabolism. The L2 level shows six genes that are significantly more abundant in the CKD group, including those involved in carbohydrate metabolism, glycan biosynthesis and metabolism, enzyme families, bosynthesis of other secondary metabolites, metabolism of other amino acids, and cellular processes and signaling, while those involved in endocrine and metabolic diseases were less common in the control group. These findings align with the serological testing results. For example, in the CKD group, serum K increased and serum Ca decreased significantly. Meanwhile, the decrease in total protein, albumin, and peripheral hemoglobin, as well as the increase in triglycerides, cholesterol, and blood glucose, suggest abnormalities in electrolyte metabolism, protein metabolism, lipid metabolism, and carbohydrate metabolism in CKD. One study indicated that a diet with a higher protein-to-fiber ratio was associated with an increased relative abundance of unclassified members of the order Oscillospirales in the gut microbiota of CKD patients. Furthermore, the intake of vegetables and whole grains was associated with *Subdoligranulum formicile*, and unclassified *Prevotella* species were associated with potatoes and foods considered to be discretionary, including sweet drinks, desserts, and animal fats. In addition, protein intake was associated with the levels of total indole sulfate and free p-cresol sulfate metabolites in the gut microbiota of patients with CKD [21]. Due to the reduced urinary potassium excretion and metabolic acidosis in CKD, hyperkalemia has become common in CKD patients. For decades, renin-angiotensin-aldosterone system (RAAS) blockers have been the main treatment for CKD. However, they are conducive to the development of hyperkalemia and trigger arrhythmia [22]. The abnormal lipid metabolism in CKD is a major factor in the development of cardiovascular disease and is a leading cause of death. Research indicates a positive correlation between the abundance of *Prevotella copri* in the gut and the aortic calcification score. Oral administration of *P. copri* can enhance the expression of Toll-like receptor 4 (TLR4), increase the level of lipopolysaccharide LPS, increase the activation of inflammatory regulatory metabolites, including Pc-LPS and NF-κB/NLRP3 signaling pathways, and promote vascular smooth muscle cell calcification, accompanied by intestinal mucosal damage [23]. Abnormal lipid metabolism in CKD leading to the development of cardiovascular disease is a major contributor to mortality. Studies have shown that providing nutritional counseling and supplementation with curcumin (Meriva) to CKD patients significantly reduced plasma levels of pro-inflammatory mediators (CCL-2, IFN-γ, and IL-4) and lipid peroxidation, while also significantly decreasing *Escherichia-Shigella* in the gut and noticeably increasing *Lachnoclostridium* [24]. This effect may be attributed to the capacity of dietary supplements to attenuate intestinal toxin accumulation, restore mucosal barrier integrity, and thereby modulate gut microbiota composition [25]. Our research confirms that patients with CKD not only experience kidney function damage but also have changes in their intestinal flora leading to dysbiosis.

Further research on the correlation between differential bacterial communities and serum markers of renal function in CKD patients revealed that while the serum uric acid level in CKD patients increased, the abundance of *Actinomyces* in the gut significantly decreased, while the abundance of *C. Solibacter* significantly increased. *Actinomyces* belong to the phylum Actinobacteria, which are mostly gram-positive bacteria capable of producing a variety of antibiotics such as streptomycin, erythromycin, and tetracycline, as well as enzyme preparations such as protease, amylase, and cellulase. They not only participate in the immune regulation and defense mechanisms of the human body, but also may play an auxiliary role in metabolic pathways [26]. For example, protease helps in the digestion of protein, decomposing large molecular proteins into small molecular peptides and amino acids which are easy for the human body to absorb and utilize. Actinobacteria can also produce metabolites such as vitamin B12 and organic acids, which are involved in the metabolism of fats, carbohydrates, and proteins, and maintain the normal function of the nervous system [27]. Some Actinomycetes can infect the human body and cause infections in the oral cavity, maxillofacial region, abdominal and pelvic cavity, and respiratory tract. For example, actinomycete infections can lead to chorioamnionitis and premature delivery [28]. *C. Solibacter* belongs to the Acidobacteriota phylum. It has the ability to adapt to and survive in acidic environments, such as acidic soil and water bodies. Moreover, it has the ability to degrade plant residue polymers, decompose organic matter, utilize carbon sources, and participate in processes such as iron cycling and monocarbon compound metabolism. G_*Candidatus* may have the potential to promote digestive peristalsis, alleviate constipation symptoms, and improve depression [29]. Research shows that the relative abundance of g_*C. Solibacter*, g_*Pseudoramibacter-Eubacterium*, g_*Peptoniphilus*, and g_*Geobacter* in elderly people with depressive symptoms is lower than that in elderly people without depressive symptoms, and the abundance of g_*C. Solibacter* is negatively correlated with PHQ-9 scores [30]. The reason may be that g_*Candidatus* is closely related to depression through purine metabolism and fatty acid metabolism [31]. Uric acid is the end product of purine metabolism, with 80% coming from the breakdown of protein and 20% from the consumption of purine-containing foods. Furthermore, 70% of its elimination occurs through the kidneys, while the remaining 30% is excreted in the feces. Since CKD patients cannot effectively eliminate various uremic toxins such as uric acid from the body due to impaired renal function, they enter the intestinal lumen, which changes the intestinal flora environment leading to dysbiosis. Dysbiotic intestinal flora can then react back on the host by producing toxins and other metabolites, inducing systemic microinflammatory reactions, which may further aggravate the degree of kidney damage [28]. The accumulation of uremic toxins in patients with CKD can lead to vascular calcification and cardiovascular disease, which is the main cause of death. This not only affects patients with advanced CKD with a GFR below 15 mL/min./1.72 m2, but also affects patients in the early stages of the disease, even in end-stage renal disease. The key uremic toxins that cause VC, namely p-cresol sulfate (PCS), indole sulfate (IS), and trimethylamine-N-oxide (TMAO), all originate from bacterial metabolism in the gut microbiota. These toxins promote VC through multiple mechanisms which include transdifferentiation and apoptosis of VSMCs, endothelial cell dysfunction, oxidative stress, interactions with the local renin-angiotensin-aldosterone system, or modification of miRNA profiles [32]. CKD patients usually need to follow the dietary principles of low protein, low phosphorus, low potassium, and low salt to reduce the burden on the kidneys and prevent complications. However, dietary habits also play a crucial role in shaping the host's gut microbiota. Previous study has shown that vegetarians exhibit significantly increased abundance of Bacteroidetes and *Prevotella* in their gut (*p* < 0.05) [33]. A cross-sectional study demonstrated that 57 CKD patients on a low-protein diet (LPD) were randomly divided into two groups receiving either probiotics or placebo. Results indicated that participants in the probiotic group exhibited a trend toward reduced microbiota-derived toxins. These findings suggest that associating probiotics with LPD may exert beneficial effects on controlling and modulating microbiota-derived, pro-atherogenic toxins in CKD patients [34]. These dietary restrictions may lead to a reduction in the types and amounts of nutrients consumed by patients. In addition, patients may also experience lipid metabolism disorders, mineral and vitamin metabolism abnormalities, and further exacerbate malnutrition. In this study, the number of *Actinomyces*, which can effectively participate in nutrient metabolism in the intestines of patients with CKD, decreased, while the number of *C. Solibacter*, which has acid resistance, increased, further indicating that intestinal flora is related to the pathological mechanism of CKD. *Actinomyces* and *C. Solibacter* may have the potential to regulate the intestinal environment in CKD.

It is noteworthy that this study has several limitations. First, the analyzed data originated from a single institution and feature a relatively limited number of cases and nonspecific staging of CKD. Second, as this was a cross-sectional study, the observed associations between gut microbiota composition and serological indicators of CKD do not imply a direct “cause-and-effect” relationship. Despite implementing rigorous inclusion criteria and stringent screening protocols during study design and case selection—such as controlling for age, sex, BMI, renal function, dietary patterns, alcohol intake, antibiotic use, and other covariates—it remains challenging to fully account for the inherent variability in clinical populations and potential residual confounding factors. To further elucidate the dynamic role of microbiota alterations in CKD progression, future investigations should employ longitudinal cohort studies integrated with multi-omics analyses, alongside animal models involving fecal microbiota transplantation (FMT) or targeted antibiotic interventions, to validate causal mechanisms and temporal relationships.

## Conclusion

The results of this study showed that there were significant differences in the composition and species abundance of the intestinal flora between patients with CKD and healthy individuals. The pathways of difference were mainly concentrated in metabolic pathways, and the abundance of *Actinomyces* and *C. Solibacter* was related to the level of blood uric acid, an indicator of renal function damage, which may be involved in the pathogenesis of CKD.

## Supplemental Materials

Supplementary data for this paper are available on-line only at http://jmb.or.kr.



## Figures and Tables

**Table 1:** Clinical data.

Group	CKD	Control	t/x^2^	*P*
Sex, male (female)	26 (16)	20 (22)	1.730	0.273
Age, year	68.81 ± 14.9	63.48 ± 10.88	1.873	0.065
BMI	23.93 ± 1.64	24.05 ± 1.41	-.356	0.723
K, mmol/l	4.41 ± 0.72	3.96 ± 0.32	3.713	0.000***
Na, mmol/l	140.74 ± 3.99	140.93 ± 2.86	-.252	0.802
Cl, mmol/l	99.38 ± 5.05	100.7 ± 2.68	-1.496	0.140
Ca, mmol/l	2.07 ± 0.22	2.24 ± 0.11	-4.698	0.000***
ALT, U/L	21.42 ± 35.76	17.74 ± 9.89	.643	0.522
AST, U/L	17.3 ± 12.61	19.63 ± 7.5	-1.028	0.307
TP, g/l	62.74 ± 8.41	68.4 ± 5.54	-3.643	0.000***
ALB, g/l	37.24 ± 7.11	41.54 ± 3.95	-3.428	0.001***
TBIL, μmol/l	9.15 ± 6.35	9.81 ± 4.19	-.556	0.580
TBA, μmol/l	5.87 ± 4.15	6.1 ± 3.1	-.289	0.773
TG, mmol/l	1.59 ± 0.71	1.2 ± 0.41	3.053	0.003***
CHO, mmol/l	4.26 ± 1.25	3.9 ± 0.69	1.634	0.107
HDL, mmol/l	1.07 ± 0.45	1.11 ± 0.31	-.500	0.619
LDL, mmol/l	2.55 ± 0.99	2.29 ± 0.8	1.343	0.183
ApoA, g/l	1.18 ± 0.33	1.25 ± 0.23	-1.174	0.244
ApoB, g/l	1.05 ± 0.24	0.81 ± 0.22	4.584	0.000***
GLU, mmol/l	5.92 ± 1.73	5 ± 0.53	3.311	0.002***
Hb, g/l	109.57 ± 25.99	132.52 ± 16.08	-4.867	0.000***

BMI, body mass index; K, potassium; Na, sodium; Cl, chloride; Ca, Calcium; ALT, alanine aminotransferase; AST, aspartate aminotransferase; TP, total protein; ALB, albumin; TBIL, total bilirubin; TBA, total bile acids; TG, triglycerides; CHO, cholesterol; HDL, high-density lipoprotein; LDL, low-density lipoprotein; ApoA, apolipoprotein A; ApoB, apolipoprotein B; Hb, hemoglobin; GLU, serum glucose. *P* < 0.05 considered statistically significant. *P* <0.05 is marked with *; *P* <0.01 is marked with **; *P* <0.001 is marked with ***.

**Table 2:** Comparison of clinical renal function indices.

Group	CKD	Control	t	df	*P*	95% Confidence interval	
						Lower limit	Upper limit
GFR	36 ± 31.09	110.29 ± 9.03	-14.872	47.872	0.000***	-84.340	-64.250
UREA	12.87 ± 7.59	4.47 ± 1.34	7.065	43.564	0.000***	6.003	10.797
CRE	330.38 ± 314.36	61.26 ± 14.33	5.542	41.170	0.000***	171.068	367.170
UA	384.76 ± 105.88	284.71 ± 66.48	5.186	82.000	0.000***	61.673	138.423
CyC	3.01 ± 1.97	0.87 ± 0.16	7.003	41.533	0.000***	1.520	2.751
β2-MG	8.33 ± 7.33	1.37 ± 0.47	6.143	41.330	0.000***	4.675	9.252

GFR, Glomerular Filtration Rate; CRE, creatinine; UA, uric acid; CyC, cystatin C ; β2-MG, blood β2-microglobulin; *P* < 0.05 considered statistically significant. If the *p*-value is less than 0.05, it is marked with *, if less than 0.01, it is marked with **, and if less than 0.001, it is marked with ***.

**Table 3:** Sample sequencing information.

Sample Name	Raw PE	Nochime	Base (nt)	AvgLen (nt)
CKD.1	104,745	62,033	25,883,260	417
CKD.2	108,666	64,587	26,870,387	416
CKD.3	113,446	68,374	28,077,351	411
CKD.4	100,153	63,815	27,155,342	426
CKD.5	104,268	65,988	27,572,378	418
CKD.6	113,125	69,674	29,014,211	416
CKD.7	112,666	69,095	28,589,293	414
CKD.8	106,699	65,121	27,042,670	415
CKD.9	103,562	61,009	25,447,019	417
CKD.10	102,287	66,368	27,609,262	416
CKD.11	116,655	69,863	29,166,687	417
CKD.12	107,948	66,456	27,553,712	415
Control 1	96,695	60,294	24,972,077	414
Control 2	103,170	64,124	26,631,933	415
Control 3	110,196	68,466	28,419,316	415
Control 4	110,644	69,654	28,871,588	415
Control 5	110,509	69,856	28,955,423	415
Control 6	106,779	66,837	27,773,006	416
Control 7	98,055	60,543	25,029,823	413
Control 8	103,729	66,559	27,592,711	415
Control 9	112,439	68,138	28,067,010	412
Control 10	108,143	65,202	26,643,428	409
Control 11	100,169	61,836	26,003,757	421
Control 12	113,405	67,598	28,187,064	417
Total	2568153	1581490	657128708	9975

Raw PE refers to the original PE reads obtained from the sequencing machine; Nochime represents the sequences that have been filtered for chimeric sequences and are ultimately used for subsequent analysis; Base denotes the number of base pairs in the final effective sequences; AvgLen is the average length of the effective sequences; CKD1-12 represent the samples from the CKD group, and Control 1-12 represent the samples from the Control group.

**Table 4:** Anosim and adonis analysis.

Comparison	Anosim		Adonis	
	R-value	*P*-value	R2	*P*r
Control -CKD	0.1724	0.003	0.09462	0.002

Anosim analysis performs a significance test for differences between groups based on the ranks of Bray-Curtis distance values, thereby determining whether the grouping is meaningful. It is conducted using the anosim function from the R vegan package. The R-value ranges between (-1, 1). An R-value greater than 0 indicates significant differences between groups, while an R-value less than 0 suggests that within-group differences are greater than between-group differences. The *P*-value represents the probability value, with *P*-value < 0.05 indicating statistical significance. ADONIS is a nonparametric multivariate variance analysis method based on Bray-Curtis distances. This method analyzes the degree to which different grouping factors explain the sample differences and uses permutation tests to assess the statistical significance of the grouping. ADONIS analysis is performed using the adonis function from the R vegan package. R2 represents the degree to which different groups explain the sample differences, *i.e.*, the ratio of group variance to total variance. A higher R2 indicates a greater degree of explanation for the differences by the grouping. *P*r represents the probability value, with *P*r < 0.05 indicating high reliability of the test.

**Table 5:** Correlation between differentiated bacteria and renal function serological markers.

Bacteria	Values	UREA	CRE	UA	β2-MG	CyC
*Faecalibacterium*	R	-0.235	-0.189	-0.224	-0.189	-0.189
	*P*	0.268	0.376	0.294	0.376	0.376
*Blautia*	R	0.092	0.086	0.042	0.135	0.113
	*P*	0.669	0.689	0.845	0.531	0.599
*Agathobacter*	R	-0.179	-0.185	-0.051	-0.224	-0.195
	*P*	0.403	0.386	0.813	0.292	0.362
*Roseburia*	R	-0.202	-0.162	-0.205	-0.136	-0.148
	*P*	0.343	0.449	0.337	0.525	0.489
*Actinomyces*	R	-0.208	-0.197	-0.426	-0.213	-0.255
	*P*	0.329	0.355	0.038*	0.317	0.229
*Candidatus_Solibacter*	R	0.334	0.307	0.461	0.294	0.309
	*P*	0.111	0.144	0.023*	0.163	0.142

R represents the correlation coefficient, with values ranging from -1 to 1. Positive values indicate a positive correlation, while negative values indicate a negative correlation. *P* < 0.05 considered statistically significant. *P* < 0.05 is marked with *.
