# Supplementary material for: Evaluation of the Correlation between Gut Microbiota and Renal Function in Chronic Kidney Disease Patients
Source: J Microbiol Biotechnol. 2025 Jun 17;35:e2502039. doi: 10.4014/jmb.2502.02039 (PMC12197812; doi:10.4014/jmb.2502.02039)
Supplement: Supplementary file 1 [file jmb-35-e2502039-supple.pdf]

J. Microbiol. Biotechnol. 2025. 35: e2502039
https://doi.org/10.4014/jmb.2502.02039

Appendix

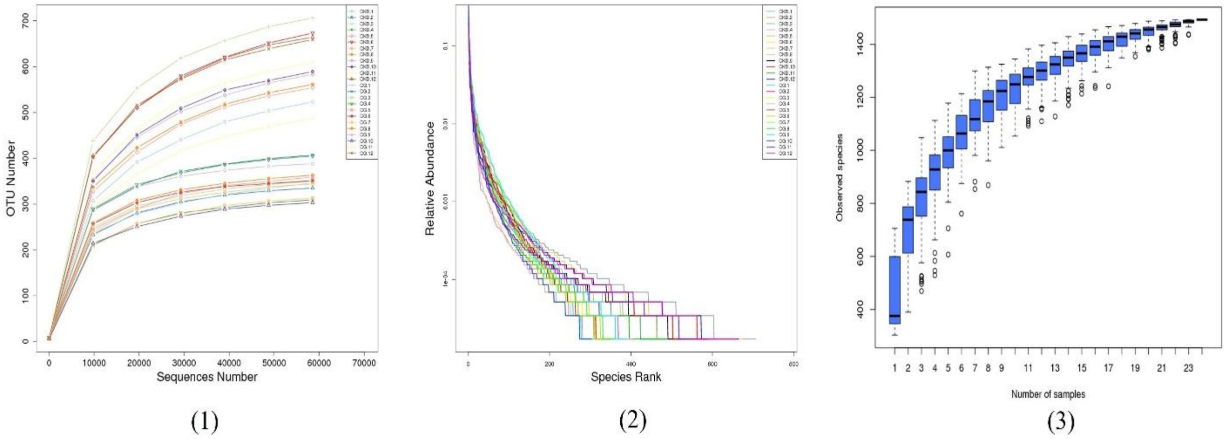

Fig. A: Supplementary graph of Alpha Diversity index. (1) Rarefaction Curve can directly reflect the rationality of the sequencing data quantity and indirectly reflect the richness of species in the sample. When the curve tends to be flat, it indicates that the sequencing data quantity is gradually reasonable. (2) Rank Abundance curve can visually reflect the richness and evenness of species in the sample. The larger the span of the curve on the horizontal axis, the higher the species richness, the flatter the curve, and the more even the species distribution. (3) Species accumulation boxplot can reflect whether the sampling is sufficient for this study. The abscissa represents the sample size, and the ordinate represents the number of species detected. As the sample size increases, the position curve of the box plot gradually becomes flatter, which means that even if the number of samples is further increased, the number of species in the environment will not significantly increase, indicating that the sampling is sufficient. The above analysis was completed using R software Version 2.15.3.
